# ‘Leading by Science’ through Covid-19: the NHS Data Store & Automated Decision-Making

**DOI:** 10.23889/ijpds.v5i4.1402

**Published:** 2021-04-07

**Authors:** MJ Mourby

**Affiliations:** Centre for Health, Law and Emerging Technologies, University of Oxford, Ewert House, Oxford, OX2 7DD, UK

**Keywords:** Covid-19 Algorithms, Automated Decision-Making, General Data Protection Regulation

## Abstract

The UK government announced in March 2020 that it would create an NHS Covid-19 ‘Data Store’ from information routinely collected as part of the health service. This ‘Store’ would use a number of sources of population data to provide a ‘single source of truth’ about the spread of the coronavirus in England. The initiative illustrates the difficulty of relying on automated processing when making healthcare decisions under the General Data Protection Regulation (GDPR). The end-product of the store, a number of ‘dashboards’ for decision-makers, was intended to include models and simulations developed through artificial intelligence. Decisions made on the basis of these dashboards would be significant, even (it was suggested) to the point of diverting patients and critical resources between hospitals based on their predictions.

How these models will be developed, and externally validated, remains unclear. This is an issue if they are intended to be used for decisions which will affect patients so directly and acutely. We have (by default) a right under the GDPR not to be subject to significant decisions based solely on automated decision-making. It is not obvious, at present, whether resource allocation within the NHS could take place in reliance on this automated modelling. The recent A Level debacle illustrates, in the context of education, the risks of basing life-changing decisions on the national application of a single equation. It is worth considering the potential consequences for the health service if the NHS Data Store is used for resource planning as part of the Covid-19 response.

## Introduction

The UK government frequently reassured us that it would be ‘led by the science’ in its response to the Covid-19 pandemic. As central government shouldered the responsibility for making potentially life-and-death choices about lockdown and critical resource allocation, this statement had a reassuring air of rigour and impartiality. And it is of course entirely appropriate that close attention is paid to the best possible epidemiological information when these choices are made.

However, scientific outputs are often at least partially automated—particularly when predictions on a national scale are attempted, and standard statistical models would take more time and human resources than is available in the early stages of a pandemic [1]. Care must be taken when we are ‘led’ by this science, so that we act upon automated calculations with an appropriate level of reliance. We have a general right under the General Data Protection Regulation (‘GDPR’) not to be subject to significant decisions based ‘solely’ on automated processing. This article considers the impact of this right on the NHS Covid-19 Data Store, and calls for greater systemic transparency in its development.

## Methods

This article draws from a review of published statements about the NHS Covid-19 Data Store, with particular reference to its use of predictive modelling for resource allocation. These statements are analysed in light of the GDPR, and Article 22 GDPR in particular, as well as associated academic commentary on the use of automated processing in decision-making. It is timely to consider automated decision-making using population-level data, given the recent backlash following the use of an equation to calculate substituted A Level grades for pupils who missed their exams due to Covid-19. It is important that sufficient safeguards are in place in the context of public health to prevent similar failures of automated decision-making.

## Background: The Data Store

The initial legal foundation for the Store was a Control of Patient Information (‘COPI’) Notice, issued by the Secretary of State to set aside the medical duty of confidence in identifiable information. This means patient information can be re-used for the Store without breach of common law confidentiality. Data protection complicance is another matter, however. While the COPI notice was issued in March 2020, it took until June for a Data Protection Impact Assessment (‘DPIA’), addressing GDPR requirements, to be made available [2].

Data Protection concerns much more than privacy. Data processing can have consequences for individuals that go beyond mere intrusion into their personal life (although this alone can be bad enough). In prohibiting solely automated decisions with legal or otherwise significant effects, Article 22 GDPR addresses the power of data processing to create actionable inferences that are made about us, from commercial marketing, to political micro-targeting and, now, how patients may be ‘diverted’ within the health system as part of a response to a global pandemic.

On 28 March 2020, Matthew Gould (the CEO of NHSX) published a co-authored post entitled *‘The power of data in a pandemic’* [3], in the Technology in the NHS blog which is designed to *‘support the Secretary of State’s vision for the NHS.’* The vision set out in this blog post is one which centralises data-based decision-making. It is explained that data from across the health and social care system—111 calls, coronavirus test results, hospital occupancy figures— will be brought together to form a *‘single source of truth’* to support decision-making.

It is evident that some degree of predictive modelling also formed part of this vision. The post stresses the importance of anticipating the demand on the health and care services. Faculty, a London-based firm specialising in Artificial Intelligence, was expected to develop:

*‘models and simulations to provide key central government decision-makers with a deeper level of information about the current and future coronavirus situation to help inform the response’*

The post indicates that a beta form of the first dashboard would be made available to government decision-makers that week. It is not clear whether that first iteration would contain models or simulations of the spread of the virus. However, the apparent aspiration to move quickly to predictive modelling in March 2020 is interesting. A UK government advisor has subsequently described the Covid-19 data relied on at the time as *‘really quite poor’* [4], raising the question as to where these models would come from and how they would be trained.

The limitations of models developed in the early stages of the pandemic were demonstrated in a systematic review and critical appraisal [5] of 31 Covid-19 prediction models, most of which had been developed on data collected between December 2019- March 2020. Owing to the nature of the pandemic at that time, the majority used data from small studies in China or Italy. The authors concluded that these models were often poorly reported and at a high risk of bias due to the limited data on which they had been developed. It has been suggested that these early models will not be useful at any stage of the pandemic due to their potential bias [6]. As of May 2020, few machine learning responses to Covid-19 appeared sufficiently mature to operationalize at scale [7].

The NHS ‘Data Store,’ by contrast, may have had better documented and more extensive data from which to develop predictive models, but we do not know the details of its modelling activities as it has thus far has been criticised for lacking transparency [8, 9]. While some steps towards transparency have been made with the release of the public-private partnership contracts [10], and the publication of the DPIA in June 2020 [11] these do not give us the full picture as to how the decision-making process works in practice. It is important to know what decision-making safeguards are in place. Writing in May 2020, Joshua Blumenstock was optimistic about the use of machine learning to target Covid-19 aid, but nonetheless advocated human guidance in model calibration, as well as to triage the *‘inevitable’* failures in automated predictions [12].

### Human Oversight in the Data Store?

It is difficult to foresee how human oversight will (or was supposed to) be applied in the NHS Data Store. Five different companies were originally mooted to be providing different components of the Store’s software. It has since been suggested that Palantir at least will only be a data processor [13], and the updated DPIA information suggests a different cast of actors are now involved [14]. Data control —the responsibility for ensuring GDPR compliance—was intended to be split between three different NHS bodies (NHSX, NHS Improvement and NHS England) [15]. As the government is posited as the ultimate decision-maker in the blog, it is also possible that the data are in fact processed on their behalf, making them joint controllers in the processing. Who is taking responsibility for overseeing this processing to gauge its reliability, and overruling its predictions where necessary?

This is an important question. Among the decisions listed in the blog which would be made on the basis of this processing are to:

- *Proactively increase health and care resources in emerging hot spots;*
- *Ensure critical equipment is supplied to the facilities with greatest need; and*
- *Divert patients/service users to the facilities that are best able to care for them based on demand, resources, and staffing capacity.*

In other words, the blog raises the potential for patients to be diverted to or away from hospitals, or staff or ventilators redeployed, on the basis of the predictions of a model developed through artificial intelligence. The mere possibility that this level of top-down control of critically ill patients might be made on the basis of automated processing from central government is a significant extension of the degree to which algorithms can be said to rule the world [16]. How does this sit with the GDPR’s general prohibition on automated processing?

## Results: Article 22 GDPR

There are a number of ways in which the Data Store can utilise automated processing in a manner compatible with Article 22 GDPR.

Firstly, there are exceptions to Article 22’s general exemption against significant decisions based solely on automated processing. Where health-related data are involved, there are only two exceptions:

1) where explicit consent is obtained (although this is unlikely to be an appropriate basis in a healthcare context [17]); 2) for reasons of substantial public interest with some basis in EU or national law [18].

The Data Protection Act 2018 does permit public authorities to use solely automated decision-making where it is a reasonable means of complying with their legal obligations [19]. In this instance, it is debatable but certainly not impossible that the NHS controllers would have such as basis. In the case of the NHS Covid-19 app, for example, the DPIA accepts it is *‘arguable’* that Article 22 GDPR applies to the app’s processing, and identifies the relevant legal powers that support any automated decision-making [20]. Similar provisions could also be said to authorise such decision-making from the Data Store.

### Meaningful Intervention in Decisions?

Secondly, Article 22 GDPR only governs decisions based ‘solely’ on automated decision making. It has been argued that this is a significant lacuna, allowing even minimal or trivial human involvement to disqualify the right [21]. Others have argued that human involvement in the decision must be substantive [22], and far more than minimal [23]. This latter interpretation has been supported by the Article 29 Working Party’s guidance, which requires that to escape the label of ‘solely automated,’ decisions must be overseen by someone within the data controller who has *‘the authority and competence to change the decision’,* and has access to all the data [24].

The Article 29 Working Party guidance is clearly aimed at decisions made by a single controller, where scientific competence and decision-making authority may rest in the same person, not in a complex and rapidly evolving joint controller undertaking. How is this to be achieved in a complex, multi-actor arrangement where five private companies, and three NHS bodies, collaborate in the production of dashboards which are presented to ‘key government decision-makers’ with an undisclosed amount of information about how much reliance can be placed on the predictions, and how well calibrated the underlying models are for the national population?

The DPIA for the Store published in June 2020 clarifies this point only to the extent of providing a blank denial. The template asks:

*Will the processing result in a decision being made about the data subject solely on the basis of automated processing?*

With a footnote adding:

*examples include the automatic refusal of an online credit application and e-recruiting practices without any human intervention*

And the simple answer given is *‘No’* [25]. This is, at least superficially, reassuring. It suggests there is no intention for significant decisions to be taken ‘solely’ on the basis of the automated processing within the Data Store. However, comments attributed to the director of artificial intelligence at NHSX suggest that the predictive elements of the NHS Covid-19 Data Store have had a dramatic effect on the management of critical resources in the NHS [26]. Automated processing evidently plays a significant role in resource-management, which is clearly a policy issue which could have critical downstream consequences for patients. It is not clear what safeguards are currently in place to ensure that human intervention in decision-making takes place, and it would be good to have this confirmed.

A potential lacuna lies in the wording of NHS England’s DPIA: *‘Will the processing result in a decision being made about **the data subject** solely on the basis of automated processing?’* This implies that Article 22 GDPR only applies if the subject of the data automatically processed is the one affected by the decision. Whereas the phrasing of Article 22 potentially casts a wider net:

*‘The data subject shall have the right not to be subject to a decision **based solely on automated processing**, including profiling, which produces legal effects concerning him or her or similarly significantly affects him or her.’*

Article 22 does not explicitly state that the automated processing in question needs to be of the data subject’s own personal data. This means it could also capture people caught by the downstream consequences of the automated processing of other people’s data. Also, it is notable that NHS England gives the examples of credit applications and e-recruiting, without reflecting on what an automated decision might look like in a resource-allocation context. Further reflection on the human/ automated interface within the Data Store might have been helpful to dispel any anxiety about automated resource allocation on the basis of population data.

Given this uncertainty, we cannot entirely rule out the possibility that automated predictions would not be subject to holistic, authoritative oversight, and the ensuing government decisions could therefore be made in sole reliance on the predictions. Meaningful intervention in the decision-making chain from automated prediction to action cannot be assumed.

### Meaningful Information?

Finally, and crucially, the main redress the GDPR offers when solely automated decisions are made is that the affected data subject is entitled to ‘meaningful information about the logic involved’ in the processing, and can challenge the decision and request human intervention. In the context of the NHS Covid-19 app, for example, Matthew Ryder QC and colleagues have advised that anyone experiencing consequences as a result of the app’s processing (e.g. who are told to remain in their home following a match with a symptomatic individual) must have the facility to challenge these automatic decisions [27]. The DPIA for the NHS Covid-19 app apparently confirms such safeguards are in place [28].

In the case of a patient diverted to or from a hospital predicted by the Store to be (in)sufficiently resourced, this safeguard is clearly ineffective. A patient suffering from an acute case of Covid-19, and who may even be close to dying, might technically be the data subject affected by the decision, but it would be appallingly inappropriate to give them ‘meaningful information’ about the logic of their triage and expect them to challenge the system that made this determination. The point at which urgent care is required is not the time to attempt a dissection of the decision which places a patient or ventilator in a particular hospital, even if it were possible to get enough information from Google, Microsoft, Palantir, Amazon, Faculty, NHS Improvement, NHS England, NHSX [29] and the government to piece together how it was made. Not all of these bodies are still involved in the Store, with new actors involved, making the demarcation of responsibility for the store even more opaque to an outsider [30].

It is vital that oversight and accountability is baked into the decision-making chain before this point is reached. It has already been argued that the scale and complexity of Big Data calls for systemic oversight, not just the exercise of individual rights [31]. This is considered further in the next section.

## Discussion: Automating Healthcare Rationing

The question of using population data for national resource-planning is interesting legally and ethically. In many ways, it can be seen as a scaled-up version of a triage protocol, in which hospitals determine which patients should be admitted to Intensive Care Units (‘ICU’), or even given ventilators (assuming a point of scarcity is reached). Machine-learning models could be used to predict who, among the inpatients in a hospital, would best benefit from ICU admission [32]. Within the ICU itself, neural networks can be used to predict in-hospital mortality more precisely [33], which could in turn influence resource allocation. At a national level, routinely collected data from across the NHS could be used to make these resourcing decisions on a larger scale. This even appears likely: comments attributed to the director of artificial intelligence at NHSX suggest that the predictive elements of the NHS Covid-19 Data Store have had a dramatic effect on the management of critical resources in the NHS [34].

This development is not necessarily unwelcome. As the above paragraph illustrates, artificial intelligence models have the potential to yield not only quicker but more accurate predictions based on large amounts of information, including multiple parameters, than manual estimates attempted at a comparable scale. But the development of such models will not be a guaranteed public good without individual and ‘systemic’ transparency [35], whereby regulators, expert communities, and patient representatives have enough information and involvement to address potential safety and ethical concerns around the processing.

The ethics of healthcare rationing at times of resource-anxiety (arguably, this is a continual concern) are fraught with the potential for bias against patients from vulnerable groups, such as older people, and those with disabilities. These decisions are often made by clinicians placed under considerable moral and psychological stress [36]. It could therefore be argued that algorithmic resource allocation at a national level is a more transparent means of adjudicating these difficult questions than forcing the pressure onto clinicians on the frontline. It has even been suggested that algorithms make their biases more readily known than a human can, and are thus (in this regard) more accountable [37].

On the surface, this is a promising prospect. If a model applied to population-level data could help ensure NHS resources are applied nationally in a way less susceptible to the ambiguous bias of human-decision-making, this could in turn support the national health inequality policy which has been advocated following longitudinal study [38]. It has been suggested that the NHS Covid-19 Data Store could, and should, support monitoring of the epidemic among BAME populations [39], which would address another dimension of health inequality, and automated modelling could well play a role in this equality monitoring.

However, the recent controversy following the outcomes of the algorithm used by Ofqual to estimate substituted A Level grades has highlighted the difficulty of basing decisions affecting so many on automated calculations. It has been reported that the equation in question was simply too sparse to accommodate all of the factors which should have been considered in calculations of this scale, complexity and importance [40]. The same argument could well be made for national resource allocation within the NHS; raising justified concerns as to whether these complex calculations can also be made using automated modelling without similar unfair or illogical consequences; which in this instance could have life-or-death ramifications (on a worst-case scenario). Furthermore, the suggestion that algorithms present an alternative to human bias has been challenged, especially when much depends on the bias within the training data [41].

### Systemic Transparency

The Information Commissioner’s Office (‘ICO’), and the National Data Guardian for Health and Social Care, should have roles to play in the Store’s design. The DPIA for the Store has a section headed ‘Advice of the ICO’ which has been left blank, leaving it unclear whether the document was indeed submitted for review [42]. The far lengthier DPIA published for the NHS Covid-19 app evidences wide consulation with the ICO (including on automated decision-making), as well as the National Data Guardian, Understanding Patient Data and the Centre for Data Ethics and Innovation [43]. The webpage is a living document which can be regularly updated as the consultation evolves; which contrasts to the signed PDF of a DPIA published for the Data Store. For all the controversy which has surrounded the NHS contact-tracing app, there have at least been high-level efforts to engage multiple experts, regulators and stakeholders in its development. This article endorses a similar approach to the development of the Data Store, which (it bears noting) is *not* voluntary for the data subjects whose information is used, and for which the effectiveness of individual data subject challenge is even more questionable.

Furthermore, to the extent that any predictive models are being used for ‘medical’ purposes (e.g. if they play a role in diagnosing patients, or inform decisions about their healthcare), the Medicines & Healthcare products Regulatory Agency and Health Research Authority should also be involved. The history of the NHS ‘streams’ app demonstratres that care should be taken around the boundary between medical and non-medical software [44], to ensure that predictive models do not accidentally evolve into unlicensed medical devices.

A combination of Article 22 GDPR, ethical and public policy reasons thus support the case for systemic, multi-stakeholder transparency around automated processing of population data; opening up automated policy decisions to expert scrutiny ahead of time.

## Conclusion

This article has queried extent to which automated decision rights should shape how we are ‘led’ by science in our response to Covid-19? From the foregoing, a number of answers emerge:

1) Although solely automated resource/ patient diversion may be lawful under the GDPR, meaningful human oversight of automated predictions should still be built in to prevent mistakes and potentially save the lives that might be affected by automated error.

2) If significant, solely automated decisions *are* made about patients, they are entitled to meaningful information about the logic of the processing involved.

3) As affected data subjects may be critically ill and effectively unable to exercise these rights in time to escape the consequences of their diversion, transparency also needs to take place at a national, ‘systemic’ level, with detailed information made publicly available, and multi-stakeholder involvement (including data protection/ health research regulators, and patient representatives). The consultation process for DPIA of the NHS Covid-19 app would be a good model to follow.

4) Making the code used in the Store’s models open source [45] is another step that could be taken in this direction.

5) To avoid confusion, details of initiatives such as the NHS Data Store should only be confirmed *after* a Data Protection Impact Assessment has taken place.

The DPIA published in June 2020 presents a radically simplified model for the Data Store, compared to the initial announcement in March 2020. NHS England is named as the sole data controller, and Palantir the only data processor [46]. Had NHSX waited until after a DPIA had been published to announce the details of the Store, many concerns could have been addressed from the outset, rather than leaving matters to the point that a legal challenge was attempted [47]. It would also avoid giving the impression that the DPIA was a kind of post-hoc justificatory exercise, when in fact a comparison of the March blogpost and the June DPIA suggest that much was done to simplify data control within the store, which provides a better prospect of accountability for data subjects. Nonetheless, the extent to which significant resource-allocation decisions are made ‘solely’ on the basis of automated processing within the store remains unclear.

The response to Covid-19 has shone a light on a new kind of use of automated predictions in healthcare. They are substantively different from the published, academic modelling of the disease which has been highly influential on government policy, but has also been publicly detailed and debated per the conventions of scientific discourse. The NHS Data Store thus raises the possibility of government reliance on models which are not subject even to the post-hoc peer review that follows publication, or externally validated as medical devices. Despite the apparent secrecy of their automated mechanisms, the spectre of life-and-death decisions being made in connection with these public-private models has been raised.

It is possible that plans for the Data Store have evolved, and there is no longer any prospect of significant decisions being made on the basis of its automated processing. Unless and until we have greater clarity as to how these models are used to support decision-making, scrutiny of the store’s lawfulness must go beyond confidentiality and the legal basis for the collection of information, and consider how much reliance is placed on automated predictions when making decisions that could critically affect patients. Such consideration needs to be systemic to adequately protect individuals.

## References

[1] Peiffer-Smadja N, Maatoug R, Lescure FX, D’Ortenzio E, Pineau J, King JR. Machine Learning for COVID-19 needs global collaboration and data-sharing. (2020) Nature Machine Intelligence. Available from: 10.1038/s42256-020-0181-6

[2] NHS England, ‘NHS COVID-19 Data Store’, available from: <https://www.england.nhs.uk/contact-us/privacy-notice/how-we-use-your-information/covid-19-response/nhs-covid-19-data-store/>

[3] Gould M, Joshi I, Tang M. The power of data in a pandemic. Technology in the NHS blog, 28 3 2020 available from: <https://healthtech.blog.gov.uk/2020/03/28/the-power-of-data-in-a-pandemic/> accessed 6 6 2020

[4] BBC News, ‘Coronavirus: Lockdown delay 'cost a lot of lives', says science adviser’ 7 June 2020. Available from: <https://www.bbc.co.uk/news/uk-politics-52955034>

[5] Wynants L, Van Calster B, Collins G, Riley R, Heinze G, Schuit E et al. Prediction models for diagnosis and prognosis of covid-19: systematic review and critical appraisal (2020) BMJ 369 :1328 10.1136/bmj.m1328 PMC7222643 32265220

[6] Hu Y, Jacob J, Parker G, Hawkes DJ, Hurst JR, Stoyanov D. The challenges of deploying artificial intelligence models in a rapidly evolving pandemic. (2020) Nature Machine Intelligence. Available from: <https://www.nature.com/articles/s42256-020-0185-2>

[7] Luengo-Oroz M, Hoffmann Pham K, Bullock J et al. Artificial intelligence cooperation to support the global response to COVID-19’ (2020) Nature Machine Intelligence. Available from: < 10.1038/s42256-020-0184-3>

[8] Ruhaak A. et al, ‘Open Letter’ 18 5 2020. Available from: <https://medium.com/@anoukruhaak/open-letter-b7cb79832064>

[9] openDemocracy. Stop the secrecy: Publish the NHS COVID data deals. Available from <https://www.opendemocracy.net/en/stop-secrecy-publish-nhs-covid-data-deals/>

[10] Lovell T. UK government releases details of COVID-19 data-sharing deals with big tech firms after legal action threat. Healthcare IT News 8 June 2020. Available from: <https://www.healthcareitnews.com/news/europe/uk-government-releases-details-covid-19-data-sharing-deals-big-tech-firms-after-legal>

[11] NHS England. Data Protection Impact Assessment: NHS COVID-19 Data Store. Available from: <https://www.england.nhs.uk/publication/data-protection-impact-assessment-nhs-covid-19-data-store/>

[12] Blumenstock J. Machine learning can help get COVID-19 aid to those who need it most. Nature 14 May 2020, available from: <https://www.nature.com/articles/d41586-020-01393-> accessed 6 6 2020 10.1038/d41586-020-01393-7 32409767

[13] Gould et al, note 3. It is only clarified that Palantir is a data processor, leaving the question of the role of other private sector actors open. The Data Protection Impact Assessment implies far fewer actors are involved in the store, suggesting plans may have evolved (see note 10).

[14] NHS England, COVID-19 Data Store, note 2.

[15] Again, as originally stated by Gould et al (note 3). This has since been contradicted/updated by the DPIA (note 10), which suggests NHS England will be the sole controller.

[16] Brkan M. Do algorithms rule the world? Algorithmic decision-making and data protection in the framework of the GDPR and beyond. (2019) 27 International Journal of Law and Technology 2

[17] Information Commissioner’s Office. When is consent appropriate? Available from <https://ico.org.uk/for-organisations/guide-to-data-protection/guide-to-the-general-data-protection-regulation-gdpr/consent/when-is-consent-appropriate/>

[18] Article 22(4)

[19] Explanatory Notes to the Data Protection Act 2018, para 115

[20] Department for Health and Social Care, ‘NHS COVID-19 app: data protection impact assessment’ 11 November 2020, available from: <https://www.gov.uk/government/publications/nhs-covid-19-app-privacy-information/the-nhs-test-and-trace-app-early-adopter-trial-august-2020-data-protection-impact-assessment#information-commissioners-advice>

[21] Wachter S, Mittelstadt B, Florid Li. Why a Right to Explanation of Automated Decision-Making Does Not Exist in the General Data Protection Regulation. (2017) 7 International Data Privacy Law 2

[22] Brkan, note 16

[23] Selbst A, Powles J. Meaningful information and the right to explanation. (2017) 7 International Data Privacy Law 4

[24] Article 29 Working Party. Guidelines on Automated individual decision-making and Profiling for the purposes of Regulation 2016/679. 3 10 2017; as last revised and adopted on 6 February 2018. Available from: <https://ec.europa.eu/newsroom/article29/item-detail.cfm?item_id=612053>

[25] DPIA, note 11

[26] Williams O. Covid-19 data store has transformed NHS resource planning, says official’ New Statesman, 9 6 2020. Available from: <https://tech.newstatesman.com/coronavirus/palantir-covid-19-data-store-nhsx>

[27] Ryder M, Craven E, Sarathy G, Naik R. COVID-19 & Tech responses: Legal opinion. 1 5 2020. Available from: <https://www.awo.agency/covid-19-legal-opinion.pdf>

[28] Department for Health and Social Care, DPIA, note 20

[29] The bodies listed by Gould et al (note 2), although subsequent reports indicate that Google is no longer participating in the data store. See Murphy M. Google dropped from NHS Covid-19 ‘Data Store’. Daily Telegraph 15 7 2020. Available from: <https://www.telegraph.co.uk/technology/2020/07/15/google-dropped-nhs-covid-19-data-store/>

[30] Downey A. NHS seeks supplier to continue Palantir’s work on Covid-19 data store. Digitalhealth 4 September 2020. Available from: <https://www.digitalhealth.net/2020/09/nhs-seeks-supplier-to-continue-palantirs-work-on-covid-19-data-store/>

[31] Vayena E, Blasimme A. Health Research with Big Data: Time for Systemic Oversight. (2018) 46 The Journal of Law, Medicine & Ethics 119 10.1177/1073110518766026 PMC6052857 30034208

[32] Cohen IG et al. The legal and ethical concerns that arise from using complex predictive analytics in health care. (2014) 33 Health Aff. 1139 10.1377/hlthaff.2014.0048 25006139

[33] Nielsen AB et al. Survival prediction in intensive-care units based on aggregation of long-term disease history and acute physiology: a retrospective study of the Danish National Patient Registry and electronic patient records. (2020) 1 Lancet Digital Health 2 10.1016/S2589-7500(19)30024-X 33323232

[34] Williams O. Covid-19 data store has transformed NHS resource planning, says official. New Statesman, 9 6 2020. Available from: <https://tech.newstatesman.com/coronavirus/palantir-covid-19-data-store-nhsx>

[35] Kaminski ME, Malgieri G. Algorithmic Impact Assessments under the GDPR: Producing Multi-layered Explanations. (2020) International Data Privacy Law (forthcoming). Available from: <https://papers.ssrn.com/sol3/papers.cfm?abstract_id=3456224>

[36] Peterson A, Largent EA, Karlawish J. Ethics of reallocating ventilators in the covid-19 pandemic BMJ 2020; 369 :m1828 10.1136/bmj.m1828 32398225

[37] Kleinberg J, Ludwig J, Mullainathan S, Sunstein CR. Discrimination in the Age of Algorithms. 5 2 2019. Available from: <https://papers.ssrn.com/sol3/papers.cfm?abstract_id=3329669.>

[38] Barr B, Bambra C, Whitehead M, Duncan WH. The impact of NHS resource allocation policy on health inequalities in England 2001-11: longitudinal ecological study. 2014 BMJ 348. Available from: < 10.1136/bmj.g3231> PMC4035504 24865459

[39] Downey A. NHS Covid-19 data store fails to collect and analyse BAME data. Digitalhealth 19 June 2020. Available from: <https://www.digitalhealth.net/2020/06/nhs-covid-19-data-store-fails-to-collect-and-analyse-bame-data/>

[40] Hern A. Ofqual's A-level algorithm: why did it fail to make the grade? The Guardian 21 August 2020. Available from: <https://www.theguardian.com/education/2020/aug/21/ofqual-exams-algorithm-why-did-it-fail-make-grade-a-levels>

[41] Barocas S, Selbst A. Big Data’s Disparate Impact. (2016) 104 California Law Review 671

[42] NHS England, DPIA, note 11

[43] Department for Health and Social Care, DPIA, note 20

[44] Lomas N. DeepMind’s first NHS health app faces more regulatory bumps. Techcrunch, 20 7 2016, available from: <https://techcrunch.com/2016/07/20/deepminds-first-nhs-health-app-faces-more-regulatory-bumps/>

[45] As Gould et al have committed to do ‘wherever possible’ in the relevant blog post (note 3). However, the involvement of multiple commercial organisations in the store casts doubt on when this will be possible.

[46] NHS England, DPIA, note 11.

[47] Lovell, note 10

